# How Anticipated and Experienced Stigma Can Contribute to Self-Stigma: The Case of Problem Gambling

**DOI:** 10.3389/fpsyg.2017.00235

**Published:** 2017-02-21

**Authors:** Nerilee Hing, Alex M. T. Russell

**Affiliations:** Experimental Gambling Research Laboratory, School of Health, Medical and Applied Sciences, Central Queensland UniversityBundaberg, QLD, Australia

**Keywords:** stigma, gambling disorder, public stigma, anticipated stigma, stereotyping, predictors, devaluation, discrimination

## Abstract

The degree to which anticipated and experienced public stigma contribute to self-stigma remains open to debate, and little research has been conducted into the self-stigma of problem gambling. This study aimed to examine which aspects of anticipated and experienced stigma (if any) predict the anticipated level of public stigma associated with problem gambling and the degree of self-stigma felt by people experiencing problem gambling. An online survey of 177 Australians experiencing problem gambling examined whether aspects of the public characterization of problem gambling, anticipated reactions to problem gamblers, and experiences of devaluation and discrimination predicted anticipated level of public stigma and self-stigma. The study found that self-stigma increases with expectations that the public applies a range of negative stereotypes to people with gambling problems, holds demeaning and discriminatory attitudes toward them, and considers them to lead highly disrupted lives. These variables directly predicted anticipated level of public stigma and indirectly predicted self-stigma. These findings lend weight to conceptualizations of self-stigma as an internalization of actual or anticipated public stigma. They also highlight the need for stigma reduction efforts, particularly those that lower negative stereotyping and prejudicial attitudes, to improve currently low rates of help-seeking amongst people with gambling problems.

## Introduction

Stigma has been identified as one of the greatest challenges facing mental health due to its debilitating effects in several life domains as affected individuals are negatively stereotyped, face social rejection, and experience prejudice and discrimination (Hogan, 2003; Hinshaw, 2006; Stuart, 2011). Self-stigma is thought to be particularly damaging, and is said to occur when individuals internalize stigmatizing social attitudes, and come to believe the negative societal conceptions and stereotypes associated with their condition (Corrigan and Watson, 2002; Watson et al., 2007; Corrigan and Rao, 2012). Whether stigma is only anticipated or directly experienced, self-stigmatizing beliefs can lead to withdrawal from social support, rejection of help, avoidance of treatment, treatment withdrawal, and limited prospects for recovery (Link and Cullen, 1990; Link, 2001; Corrigan, 2004; Vogel et al., 2007). Negative impacts have also been found on psychological well-being, life satisfaction, goal-related behavior, social adjustment, and social and economic opportunities such as employment, income, housing, and social relationships (Markowitz, 1998; Perlick et al., 2001; Corrigan, 2004; Vogel et al., 2007; Corrigan et al., 2009). In fact, the self-stigma associated with mental illness has been viewed by some as equally or more debilitating than the illness itself (Corrigan, 1998; Vogel et al., 2006).

Labeling theory (Scheff, 1966) provides one explanation for the damaging effects of stigma. It posits that the labeling of individuals as mentally ill confers a deviant status that is stigmatized through rejection, devaluation, isolation and discrimination. This causes eventual adoption of a deviant self-concept and further aberrant behavior aligned with others’ (lowered) expectations of how mentally ill people should behave. As such, labeling theory views stigma as the direct cause of sustained mental illness (Markowitz, 1998). Modified labeling theory (Link, 1987; Link et al., 1989) is a similar but less extreme perspective. Being aware of the negative stereotypes associated with a stigmatized condition, affected individuals anticipate devaluation and discrimination, and adapt their behavior to avoid, challenge or otherwise cope with expected social rejection. These responses act to further limit social networks and opportunities, thus indirectly sustaining mental illness (Markowitz, 1998). A variant of modified labeling theory is the ‘why try’ effect where self-stigma is viewed as interfering with achievement of life goals, not only through avoiding situations where social rejection is anticipated, but also because diminished self-esteem and self-efficacy leave people unable to tackle the requirements for achieving these goals (Corrigan et al., 2009). Labeling theory and its variants share the assumption that classifying individuals as mentally ill can change their self-concept and social identity, fuelling a self-fulfilling prophecy as self-esteem, self-efficacy and social functioning are diminished.

Stereotype threat has also been proposed as a mechanism that leads to underperformance and perhaps to self-stigmatization. Stereotype threat occurs when individuals belonging to a negatively stereotyped group feel threatened or anxious in situations where they risk confirming, as self-characteristic, a negative stereotype about their group (Steele and Aronson, 1995). It is situation dependent, that is, felt in circumstances where one can be judged by, treated according to, or self-fulfill negative stereotypes about one’s group (Spencer et al., 1999). Stereotype threat has been examined in relation to the intellectual performance of women (Spencer et al., 1999; Schmader, 2002), African Americans (Steele and Aronson, 1995; Aronson et al., 2002), and students from low socioeconomic backgrounds (Croizet and Claire, 1998). Overall, these experimental studies have found that stereotype threat leads to underperformance because the apprehension induced when salient stereotypes are primed disrupts or distracts from the intellectual task at hand. Less is known about the longer-term effects of stereotype threat, although Steele (1990) proposed that the resulting anxiety can lead to internalization of inferiority (self-stigma), which may manifest as adopting a victim identity, assigning blame to others, or underutilizing available opportunities.

However, the preceding views of stigma are not universally accepted. Researchers have questioned whether and how much of the commonly espoused negative outcomes are due to anticipated and experienced public stigma, as opposed to other causal factors. One argument asserts that social rejection arises because of the symptoms of the mental illness itself, and that being officially labeled as mentally ill is relatively inconsequential and enables access to beneficial treatment (Gove and Fain, 1973; Chauncey, 1975; Gove, 1982; Weinstein, 1983). Another view is that the shame arising from self-stigma may not be due to anticipated or experienced stigma, but due to a failure to meet one’s own standards, values and ideals; that is, shame may not always have a social component but might arise from self-judgment alone (Aldrich, 1939; O’Hear, 1976; Kekes, 1988; Fortenberry et al., 2002). Research has also identified that, while some individuals experience diminished self-esteem in response to stigma, some instead react with anger, and others appear to completely ignore it Corrigan and Watson (2002). Thus, public stigma may not always negatively impact on people with mental illness nor lead to self-stigma. Overall, the degree to which anticipated and experienced public stigma contribute to self-stigma remains open to debate. Further, the relative contribution of specific aspects of public stigma to self-stigma is not well-understood across the diversity of mental health conditions. The public stigmatization of some conditions more than others (Pescosolido et al., 1999; Feldman and Crandall, 2007) is likely reflected in varying degrees of self-stigma felt by different stigmatized groups.

This paper focuses on a condition receiving little attention in stigma research – problem gambling, a behavioral addiction referred to as gambling disorder in the DSM-V (American Psychiatric Association, 2013). Problem gambling is characterized by difficulties in limiting money and/or time spent on gambling which leads to adverse consequences for the gambler, others, or for the community (Neal et al., 2005). Very few studies have examined the self-stigma associated with problem gambling. Horch and Hodgins (2015) found that stereotype agreement (agreement that negative stereotypes were true of people with problem gambling) was strongly associated with self-stigma in a sample of 155 problem gamblers. However, their major focus was on the effects of self-stigma on self-esteem, shame, coping and treatment-seeking, rather than on contributors to self-stigma itself. Hing et al. (2016a) qualitatively explored how people with gambling problems may internalize a range of self-stigmatizing beliefs. While anticipated public stigma appeared to shape self-stigma, some participants described how these beliefs also emanated from self-judgment and the dissonance between their current (problem-saturated) and idealized (problem-free) identity. The authors therefore questioned whether self-stigma is always an internalization of anticipated public stigma, or instead can arise because the problem gambling behavior violates personal values and desired self-concept. In their qualitative study involving 30 participants, Carroll et al. (2013) also queried whether the source of the shame accompanying problem gambling is primarily external or internal. Conducting a quantitative examination of the extent to which anticipated and experienced stigma predict self-stigma in relation to problem gambling can help to address this question.

**FIGURE 1:**
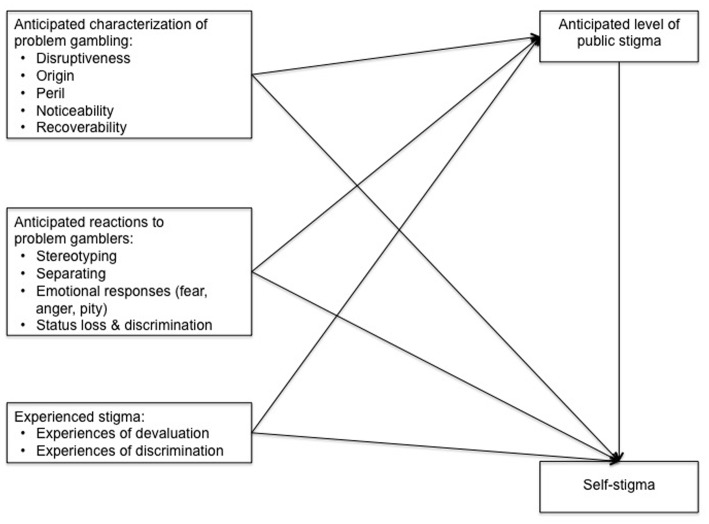
**Research model**.

The current study therefore aimed to examine which aspects of anticipated and experienced stigma (if any) predict the anticipated level of public stigma associated with problem gambling and the degree of self-stigma felt by people experiencing problem gambling. Below we present our research model (**Figure 1**) and explain its key constructs and relationships.

### Anticipated Characterization of Problem Gambling

If self-stigma arises from internalizing actual or anticipated public attitudes to the stigmatized condition (Corrigan and Rao, 2012), several aspects of public stigma can be expected to contribute to self-stigma. One aspect is the public characterization of the stigmatized condition. Prominent theories suggest that conditions perceived to be an individual’s own fault (Weiner et al., 1988), dangerous to others (Corrigan et al., 2003), and non-recoverable, highly disruptive and difficult to conceal (Jones et al., 1984), attract more public stigma. In relation to problem gambling, Hing et al. (2016b) found that predictors of stronger public stigma are beliefs that the condition is caused by bad character, is perilous, non-recoverable, disruptive and noticeable. University students considered stressful life circumstances and bad character as the main causes of problem gambling, attributed substantial levels of responsibility for the problem to the gambler, and the level of stigma they assigned increased with perceptions of dangerousness (Horch and Hodgins, 2008; Dhillon et al., 2011). Perceiving their condition to be characterized in such highly stigmatizing ways as these may then increase self-stigma amongst those with gambling problems.

### Anticipated Reactions to Problem Gamblers

Stigmatizing reactions to a condition, such as stereotyping, separating, negative emotional responses, and status loss and discrimination (Link et al., 2004), may also affect the nature and severity of anticipated public stigma and associated self-stigma. Corrigan et al. (2006) found that stereotype agreement was associated with self-concurrence (believing the negative stereotypes apply to oneself) and decrements in self-esteem amongst people with psychiatric disabilities. Horch and Hodgins (2015) also found that stereotype agreement was strongly related to self-stigma in relation to problem gambling, and that a range of negative stereotypes are commonly applied to those with the condition (Horch and Hodgins, 2013). Several studies have revealed substantial social distancing (separating) from people with gambling problems (Feldman and Crandall, 2007; Horch and Hodgins, 2008; Dhillon et al., 2011; Hing et al., 2016b). Another revealed that most people devalue (assign status loss) and discriminate against problem gamblers, and tend to react to them with both anger and pity (Horch and Hodgins, 2008). Research has consistently found that the more people believe that others devalue their group (i.e., consider them of lower social status), the worse their reported psychological well-being (Quinn and Chaudoir, 2009). Thus, several types of anticipated reactions to problem gamblers are included in our model, given their likelihood of increasing both anticipated public stigma and self-stigma amongst people with gambling problems.

### Experienced Stigma toward Problem Gamblers

The severity of self-stigma may also depend on whether the associated public stigma is only anticipated or is experienced through discriminatory actions and demeaning attitudes (Corrigan and Rao, 2012). Individuals with noticeable or disclosed stigmatized conditions typically encounter a range of degrading and discriminatory actions from others, including being treated as less competent, being told to lower their life expectations, being shunned or avoided, and being denied employment, volunteer work and health insurance (Wahl, 1999). However, less experienced stigma is reported by those who can conceal a condition (Quinn and Chaudoir, 2009; Hing et al., 2016a). Markowitz (1998) found that actual experiences of discrimination had negative effects on life satisfaction and psychological well-being amongst people with mental illness, additional to those explained by anticipated stigma. Thus, we have included experienced stigma in our model predicting both anticipated level of public stigma and self-stigma.

### Anticipated Level of Public Stigma Assigned to Problem Gambling

Anticipated stigma has been associated with heightened psychological distress, especially for concealable conditions (Quinn and Chaudoir, 2009). Thus, perceptions of stigmatization can have deleterious effects on mental health even when experienced stigma may be rarely experienced due to the hidden nature of a condition. This is because those affected are typically aware of associated stereotypes, may witness disparaging comments or actions toward the stigmatized group, and may see hurtful media portrayals of those with their condition (Wahl, 1999). In relation to problem gambling, the actual level of public stigma has been found to be a function of how the stigmatized condition is characterized and the responses it elicits, as discussed above. However, no prior research has examined what determines the global level of public stigma anticipated by individuals with gambling problems. Nevertheless, based on their association with actual public stigma, we have included anticipated characterization of problem gambling, anticipated responses to it, and experiences of experienced stigma as predictors in our model.

### Self-Stigma of Problem Gambling

Diminished self-esteem, self-efficacy and perceived social worth typically accompany self-stigma and manifest as feelings of shame, guilt, inadequacy and weakness (Corrigan, 2004; Watson et al., 2007). Qualitative research has revealed substantial self-stigma amongst people with gambling problems, expressed as feeling ashamed, embarrassed, stupid, weak, bad and worthless (Carroll et al., 2013; Hing et al., 2016a). While the effect of anticipated public stigma on the self-stigma of this group has not previously been investigated, anticipated public stigma has been consistently linked to deleterious psychological outcomes amongst those with mental disorders (Mak et al., 2007). Thus, our model depicts self-stigma as being directly impacted by public stigma, and indirectly by the independent variables in the model.

### Summary

Our model examines the direct effects of each of our independent variables on anticipated (global) level of public stigma and on self-stigma, and the indirect effects of the independent variables (via anticipated public stigma) on self-stigma. This represents new research in the gambling studies field with potential to improve our understanding of contributors to self-stigma amongst people with gambling problems. Because there is no universal agreement about the source of self-stigma for mental health problems, and because foundational research into predictors of self-stigma associated with problem gambling is lacking, no hypotheses are proposed. Instead, this should be considered an exploratory study of relationships between self-stigma, public stigma and anticipated and experienced stigma in relation to problem gambling.

## Materials and Methods

### Recruitment and Participants

We surveyed an Australia sample of people who had experienced problem gambling in the past 12 months, assessed as having a score of 8 or more on the Problem Gambling Severity Index (PGSI; Ferris and Wynne, 2001). Two recruitment methods were used: e-mailing an existing database of gamblers who had previously completed our surveys, who consented to being recontacted and who met the criteria; and through Google advertisements. Both recruitment methods provided an anonymous link to our online survey, which ran from May–July 2014. Participants were offered a AU$20 shopping voucher for completing the survey.

We identified and emailed 395 eligible respondents from our database. Thirty-six emails bounced back and we received 117 completed responses, for a response rate of 32.6%. Because our target sample was approximately 200 participants, we then commenced Google advertisements which ran for 3 weeks and gained an additional 86 responses, for a total of 203 completed responses from 351 potential respondents who started the survey (completion rate = 57.8%). Median completion time was 27.5 min. Both the recruitment email to respondents from our database and the Google recruitment advertisement invited participation from people ‘who have experienced a gambling problem.’ The survey did not ask how participants had heard about the study, so we could not compare their responses, but our recruitment notices meant that only individuals who had self-acknowledged a gambling problem were included. Additionally, only individuals scoring as problem gamblers on the PGSI were included in the analysis, with 26 respondents excluded for this reason. The final sample for analysis of 177 respondents was mostly male (66.5%), with a mean age of 40.3 years (*SD* = 13.8). Most of the sample (91.6%) reported speaking English as their main language at home.

### Measures

#### *Problem Gambling Severity Index* (PGSI: Ferris and Wynne, 2001)

The PGSI is a widely used and validated measure of problem gambling status. The PGSI was developed specifically for use in estimating the prevalence of problem gambling at the population level and is the preferred measure for prevalence studies in Australia and numerous other countries, due to the scale’s excellent reliability, dimensionality, external/criterion validation, item variability, practicality, applicability and comparability (McMillen and Wenzel, 2006; Holtgraves, 2009; Orford et al., 2010). The PGSI contains nine questions with response categories scored as ‘Never’ = 0, ‘Sometimes’ = 1, ‘Most of the time’ = 2, and ‘Almost always’ = 3. These are summed for a score between 0 and 27, where 0 = non-problem gambler; 1 or 2 = low risk gambler; 3 to 7 = moderate risk gambler; and 8 or more = problem gambler. Cronbach’s alpha for the PGSI in this sample was 0.91.

#### Self-Stigma

Themes from Carroll et al. (2013) were analyzed to create 19 questions measuring participants’ negative emotional reactions to their gambling. Participants rated how strongly they agreed or disagreed that their gambling has made them feel, for example, ‘ashamed,’ ‘stupid,’ ‘inadequate,’ and ‘weak.’ Response options were ‘Strongly disagree’ (0), ‘Disagree’ (1), ‘Neither agree nor disagree’ (2), ‘Agree’ (3), and ‘Strongly agree’ (4). Cronbach’s alpha for the Self-Stigma Scale in this sample was 0.95.

#### Anticipated Level of Public Stigma

This was measured by asking participants how much stigma they think most people attach to problem gambling. Response options were ‘None’ (0), ‘A small amount’ (1), ‘A moderate amount’ (2), ‘A large amount’ (3), and ‘An extreme amount’ (4).

#### Anticipated Characterization of Problem Gambling: Disruptiveness, Origin, Peril, Noticeability, Recoverability

Based on Jones et al.’s (1984) dimensions of stigmatized conditions, these variables were captured by asking ‘How strongly do you think MOST PEOPLE would do the following?’: ‘Think that being a problem gambler disrupts the person’s life’ (disruptiveness); ‘Think that becoming a problem gambler is the person’s own fault’ (origin); ‘Think that problem gamblers are likely to do something violent to other people’ (peril); ‘Would notice if a close friend was a problem gambler’ (noticeability); and ‘Think that people can recover from being a problem gambler’ (recoverability). Response options were ‘Strongly disagree’ (0), ‘Disagree’ (1), ‘Neither agree nor disagree’ (2), ‘Agree’ (3), and ‘Strongly agree’ (4).

#### Anticipated Reactions to Problem Gamblers: Stereotyping, Separating, Fear, Anger, Pity, Status Loss, and Discrimination

To measure stereotyping, 16 items were presented, based on common stereotypes of problem gamblers identified in the literature (Carroll et al., 2013; Horch and Hodgins, 2013; Hing et al., 2014). Examples include social – anti-social, rational – irrational and open – secretive. Participants were asked to indicate how much they thought that most people believed each listed characteristic applied to problem gamblers on a seven-point semantic differential scale. Response options were coded from 0 to 6 with higher scores indicating greater endorsement of the negative stereotype. Cronbach’s alpha for our Stereotyping Scale in this sample was 0.89.

For the remaining variables (i.e., all other reactions apart from stereotyping), the stem question was ‘How strongly do you think MOST PEOPLE would do the following?,’ followed by: ‘Would not want to interact with a problem gambler” (separating); ‘Would be afraid of a problem gambler’ (fear); ‘Feel that problem gamblers make them angry’ (anger); ‘Would feel sorry for a problem gambler’ (pity); and ‘Would look down upon problem gamblers’ (status loss and discrimination). Response options were ‘Strongly disagree’ (0), ‘Disagree’ (1), ‘Neither agree nor disagree’ (2), ‘Agree’ (3), and ‘Strongly agree’ (4).

#### Experiences of Devaluation

Devaluation was measured by nine items adapted from Kessler et al. (1999) capturing general devaluation experiences due to gambling. Respondents were asked: ‘How often have you experienced each of the following because someone *thought* you had a gambling problem?,’ e.g., ‘treated as if you are inferior,’ ‘insulted or called names.’ Responses options were ‘Never’ (0), ‘Rarely’ (1), ‘Sometimes’ (2), and ‘Often’ (3). Cronbach’s alpha for the Devaluation Scale in this sample was 0.93.

#### Experiences of Discrimination

Discrimination was measured by 13 items adapted from Kessler et al. (1999). Respondents were asked: ‘Have you ever been discriminated against in the following ways because people thought you had a gambling problem?,’ e.g., ‘fired from a job,’ ‘denied a bank loan,’ ‘prevented from renting somewhere to live.’ Response options were ‘Yes’ (1) and ‘No’ (0). Cronbach’s alpha for the Discrimination Scale in this sample was 0.79.

#### Demographics

Age (in years), gender (male/female) and main language spoken at home were asked, with the latter being an indicator of ethnicity.

### Ethics

This study was carried out in accordance with the recommendations of the National Statement on the Ethical Conduct of Research Involving Humans, administered by XX University Human Research Ethics Committee (name withheld for anonymity) with written informed consent from all subjects. All subjects gave written informed consent in accordance with the Declaration of Helsinki. Because of the sensitive nature of the survey and the vulnerability of the sample, an informed consent preamble warned that some questions were confronting and challenging, assured respondents of confidentiality and anonymity, and advised that respondents could withdraw their participation at any time. Each page of the survey contained contact details for Lifeline and for telephone and online gambling help services.

### Analysis

The data were analyzed using a combination of SPSS v23 for Mac and AMOS v23 for Windows. All variables were initially correlated using pairwise correlations, and means and standard deviations were calculated for each variable. Skewness was assessed by examining skewness and standard error of skewness data for each variable. While some skewness was evident for some variables, the pairwise correlations did not differ markedly whether we used Pearson’s or tau-b correlations. For example, all variables that were significantly correlated with self-stigma using Pearson’s correlations were also significant using Kendall’s tau-b. Then the relationship between each of the 13 possible predictors of self-stigma, including tests for mediation by anticipated level of public stigma, were analyzed separately using the PROCESS macro for SPSS (Hayes, 2013), while controlling for age, gender and PGSI score of the respondent.

As the mediation analyses may not have been independent results, the statistically significant mediation models were combined into an overall model, to control for the possible effects of each of the other predictors on the mediator (anticipated level of public stigma) and dependent variable (self-stigma). This analysis was run in AMOS v23, using bias-corrected bootstrapped confidence intervals to understand the nature of the indirect effects. Alpha was set at 0.05 for all analyses unless stated otherwise. As the survey was forced response for all questions, there were no missing data.

## Results

### Bivariate Correlations

Pearson’s correlations were calculated between all variables of interest. Amongst the predictors (**Figure 1**), anticipated level of stigma, and the independent variables of disruptiveness, origin, stereotyping, separating, anger, and status loss and discrimination, were significantly positively correlated with self-stigma. Pity was negatively and weakly correlated with self-stigma, so was not considered in additional analyses.

Anticipated level of public stigma was considered to be a possible mediator and thus the correlations between each of the predictors and this variable were also examined. The independent variables of anticipated disruptiveness, origin, stereotyping, separating, anger, and status loss and discrimination were found to be positively correlated with anticipated level of public stigma. Anticipated peril to others was also correlated, but very weakly so was not considered further.

Finally, moderate positive correlations were observed between some of the independent variables (**Table 1**).

**Table 1:** Raw correlations between variables, with mean and SD for each variable (*N* = 177).

Variable	1	2	3	4	5	6	7	8	9	10	11	12	13	14	15	16	17	18
Self stigma (1)	1																	
Public stigma (2)	0.38^∗∗∗^	1																
Stereotyping (3)	0.31^∗∗∗^	0.34^∗∗∗^	1															
Disruptiveness (4)	0.19^∗^	0.26^∗∗∗^	0.19^∗^	1														
Origin (5)	0.27^∗∗∗^	0.22^∗∗^	0.46^∗∗∗^	0.08	1													
Separating (6)	0.26^∗∗∗^	0.25^∗∗^	0.37^∗∗∗^	0.18^∗^	0.30^∗∗∗^	1												
Anger (7)	0.20^∗∗^	0.20^∗∗^	0.22^∗∗^	0.18^∗^	0.12	0.35^∗∗∗^	1											
SLD@ (8)	0.27^∗∗∗^	0.33^∗∗∗^	0.46^∗∗∗^	0.16^∗^	0.57^∗∗∗^	0.47^∗∗∗^	0.28^∗∗∗^	1										
Devaluation (9)	0.03	-0.06	0.06	-0.08	-0.10	0.08	0.17^∗^	-0.01	1									
Discrimi-nation (10)	-0.10	-0.05	-0.08	-0.14	0.02	-0.08	-0.05	0.02	0.29^∗∗∗^	1								
Recover-ability (11)	-0.03	-0.13	-0.09	0.04	-0.02	-0.12	0.06	-0.12	0.08	-0.08	1							
Noticeability (12)	-0.04	0.02	-0.03	0.29^∗∗∗^	-0.26^∗∗^	0.01	0.06	-0.15	0.12	0.05	0.12	1						
Peril to others (13)	0.10	0.15^∗^	0.19^∗^	0.14	0.05	0.29^∗∗∗^	0.28^∗∗∗^	0.21^∗∗^	0.28^∗∗∗^	0.10	-0.15^∗^	0.17^∗^	1					
Fear (14)	0.13	0.09	0.11	0.08	0.05	0.32^∗∗∗^	0.21^∗∗^	0.23^∗∗^	0.18^∗^	0.04	0.03	0.13	0.44^∗∗∗^	1				
Pity/helping (15)	-0.16^∗^	-0.04	-0.17^∗^	0.14	-0.29^∗∗∗^	-0.16^∗^	-0.05	-0.23^∗∗^	0.06	0.10	0.18^∗^	0.24^∗∗^	0.00	0.12	1			
Gender ˆ (16)	0.31^∗∗∗^	0.15^∗^	0.20^∗∗^	0.02	0.17^∗^	-0.03	0.06	0.10	-0.11	-0.05	0.14	-0.05	-0.09	0.06	-0.16^∗^	1		
Age (17)	0.18^∗^	0.00	0.02	-0.03	0.02	0.01	0.03	-0.05	-0.19^∗^	-0.15^∗^	0.13	0.11	-0.13	-0.03	-0.10	0.36^∗∗∗^	1	
PGSI (18)	0.40^∗∗∗^	0.06	0.15^∗^	0.03	0.09	0.11	0.16^∗^	0.10	0.16^∗^	0.26^∗∗^	-0.11	0.00	0.12	-.01	-0.16^∗^	0.12	-0.1	1
Mean	3.08	2.99	5.46	3.14	3.16	0.24	2.37	2.84	1.09	0.07	2.48	1.97	1.51	1.75	1.94	0.33	40.33	17.04
*SD*	0.62	0.90	1.00	0.80	0.96	1.1	1.04	1.18	0.78	0.13	0.98	1.13	1.03	1.06	1.22	0.47	13.79	5.43
*N*	177	177	177	177	177	177	177	177	177	177	177	177	177	177	177	177	177	177

*Pearson’s correlations. ˆGender coded as 0 = m, 1 = f. All scales range between 0 and 4 (except stereotyping from 0–6 and PGSI from 8–27). @SLD = status loss and discrimination. ^∗^*p* < 0.05, ^∗∗^*p* < 0.01, ^∗∗∗^*p* < 0.001*.

### Individual Mediation Analyses

Mediation analyses were conducted to determine whether the relationship between each of the predictors and self-stigma was mediated by anticipated level of public stigma. Variables were not considered for mediation analysis if they were not significantly and positively correlated with both self stigma and anticipated level of public stigma. Thus, the predictors included were: stereotyping, disruptiveness, origin, separating, anger, and status loss and discrimination. All analyses controlled for age, gender and PGSI score, with results shown in **Table 2**.

**Table 2:** Regression coefficients for tests of the effects of each predictor on self-stigma, including tests of mediation through public stigma, controlling for age, gender and PGSI score (*N* = 177).

Predictor	Effect on public stigma (mediator)				Direct effect on self-stigma				Indirect effect on self-stigma		Full Model R2
	b (SEb)	*t*	*p*	95% CI (LL:UL)	b (SEb)	*t*	*p*	95% CI (LL:UL)	b (SEb)	95% CI (LL:UL)ˆ	
Stereotyping	**0.29 (0.07)**	**3.25**	**0.001**	**0.58:2.38**	0.07 (0.04)	1.81	0.072	-0.01:0.16	**0.06 (0.02)**	**0.03:0.10**	0.36
Disruptiveness	**0.29 (0.08)**	**3.53**	**0.001**	**0.13:0.45**	0.08 (0.05)	1.54	0.130	-0.02:0.17	**0.06 (0.02)**	**0.02:0.12**	0.36
Origin	**0.18 (0.07)**	**2.59**	**0.011**	**0.04:0.32**	**0.09 (0.04)**	**2.25**	**0.030**	**0.01:0.17**	**0.04 (0.02)**	**0.01:0.08**	0.37
Separating	**0.21 (0.06)**	**3.45**	**0.001**	**0.09:0.33**	**0.09 (0.04)**	**2.50**	**0.013**	**0.02:0.16**	**0.04 (0.02)**	**0.02:0.08**	0.38
Anger	**0.17 (0.06)**	**2.58**	**0.011**	**0.04:0.29**	0.04 (0.04)	1.05	0.294	-0.03:0.11	**0.04 (0.02)**	**0.01:0.08**	0.36
SLD#	**0.17 (0.06)**	**2.58**	**0.011**	**0.04:0.29**	0.04 (0.04)	1.05	0.294	-0.03:0.11	**0.04 (0.02)**	**0.01:0.08**	0.36

*ˆ The confidence interval for the indirect effect was calculated using bias corrected bootstrapping with default 1,000 draws. #SLD = Status loss and discrimination. Significant effects shown in bold*.

Public stigma either completely or partially mediated the relationship between the predictor and self-stigma. This indicates that self-stigma is not driven directly by stereotyping, disruptiveness, anger or status loss and discrimination, but by anticipated public stigma, which is in turn driven by these variables. Origin and separating drive self-stigma both directly and indirectly through public stigma.

### Multivariate Mediation Model

**FIGURE 2:**
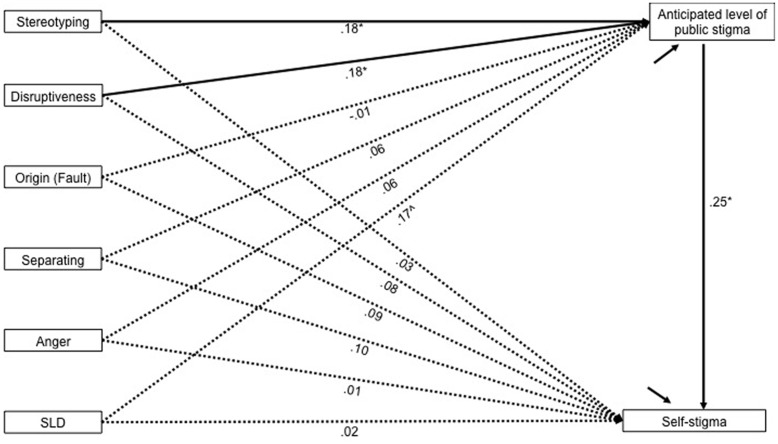
**Path model indicating mediation effects of public stigma on the relationship between the predictors and self-stigma**. The bold paths are statistically significant, while dashed paths are not (α = 0.05). Standardised coefficients are presented. ^∧^The path from SLD to perceived public stigma is not statistically significant (*p* = 0.077), however the indirect effect to self-stigma is significant. Indirect effects for stereotyping and disruptiveness are also statistically significant (*p* < 0.05). The model also includes age, gender and PGSI as control variables, although these are not shown here in the interests of clarity. ^∗^*p* < 0.05, ^∗∗^*p* < 0.01, ^∗∗∗^*p* < 0.001.

Because there may be overlap between the individual mediation analysis results, an overall model was analyzed using AMOS and depicted in **Figure 2**. Age, gender and PGSI score were controlled for. Predictors included were the same variables included in the individual mediation analyses.

The model was analyzed using bias-corrected bootstrapping (default 10,000 draws) to estimate the indirect effect of each predictor on self-stigma. As the model was just-identified, model fit statistics were not computed, although this still allows for testing of specific paths within the model (Tabachnick and Fidell, 2014, p. 764). When controlling for all other variables, including age, gender and PGSI score, the only significant predictors of anticipated level of public stigma, and indirectly of self-stigma, were stereotyping and disruptiveness (**Figure 2**). A significant indirect effect of status loss and discrimination was also observed, although the path from status loss and discrimination to anticipated level of public stigma was not statistically significant (*p* = 0.077). This most likely reflects lack of power and a larger sample may have yielded a significant path. Indeed we would have preferred a larger sample for this final analysis, but people with gambling problems are not particularly prevalent in the population and are therefore expensive to recruit.

The coefficients in the model also indicate that none of the predictors have direct effects on self-stigma when an indirect path through anticipated level of public stigma is available, and that origin, as well as separating and anger, do not have unique effects on anticipated level of public stigma, or indirectly on self-stigma.

## Discussion

The results of this study indicate that the self-stigma of problem gambling is directly associated with anticipated level of public stigma, at least in our study sample. That is, respondents with higher self-stigma were more likely to perceive the associated public stigma as more severe, and vice versa. One interpretation of this finding is that anticipated public stigma is a key determinant of the self-stigma commonly reported by people with gambling problems (Carroll et al., 2013; Horch and Hodgins, 2015; Hing et al., 2016a). This interpretation supports the contention that self-stigma at least partly arises from an internalization of anticipated public attitudes toward problem gambling, as widely suggested in relation to mental illness generally (Corrigan and Watson, 2002; Corrigan and Rao, 2012) and to concealable stigmatized conditions in particular (Wahl, 1999; Quinn and Chaudoir, 2009). Further, research has consistently found that anticipated public stigma for mental illness has damaging psychological effects on the stigmatized group (Mak et al., 2007). Previous research has found that problem gambling is heavily stigmatized (Feldman and Crandall, 2007; Horch and Hodgins, 2008; Hing et al., 2016b), so it is highly likely that people experiencing problem gambling are aware of these negative societal attitudes and beliefs, which they then apply to themselves. In turn, this interpretation lends weight to calls for stigma reduction strategies for problem gambling (Hing et al., 2016a,b) to lower self-stigma and encourage problem acknowledgment, disclosure and help-seeking which are currently at very low rates (Cunningham, 2005; Hing et al., 2016c). However, the degree of public stigma toward problem gambling cannot be the only determinant of self-stigma, given the association was only moderately strong and given that the latter varies amongst those with gambling problems. Research into mental illness has also revealed substantial variability in how people cope with and respond to specific stigmatized identities (Quinn and Chaudoir, 2009). Further research is needed into individual difference variables which may influence the extent to which public stigma is internalized as self-stigma in relation to problem gambling.

An alternative explanation for the association between self-stigma and anticipated level of public stigma is that having higher self-stigma leads to perceptions that problem gambling is heavily stigmatized by most people. Some evidence exists that people experiencing problem gambling perceive its public stigmatization to be more severe than it actually is, rating it as more stigmatized than alcoholism and schizophrenia when the reverse was found in the general population in the same jurisdiction (Hing, 2016). A qualitative interview study revealed examples of people receiving much more positive and supportive responses than they expected when they finally disclosed their gambling problem to others (Hing et al., 2016a). These limited findings suggest the potential value of comparative studies between actual and anticipated levels of public stigma toward problem gambling. If the former is lower than the latter, educating gamblers that the public is less judgmental than they anticipate could also lower self-stigma and encourage disclosure and help-seeking. The association of higher self-stigma with higher anticipated level of public stigma indicates the potential value of longitudinal research to clarify causal directions.

Our study also found that anticipated level of public stigma increased with stronger beliefs that most people apply a range of negative stereotypes to problem gamblers and consider that being a problem gambler disrupts the person’s life. These were the only significant predictors when all other variables were controlled for, including age, gender and PGSI score. They were also significant indirect predictors of self-stigma (via anticipated level of public stigma), along with anticipated status loss and discrimination. Thus, anticipated level of public stigma, and in turn self-stigma, appear to be driven by beliefs that the public generally considers problem gamblers to have a range of negative attributes, to lead disrupted lives and to be inferior in status. These appear to be the first published findings identifying predictors of anticipated public stigma of problem gambling from the perspective of people experiencing the condition. Previous research has identified these same predictors, amongst others, but only from the perspective of the general population (Hing et al., 2016c,d). This general population sample applied a range of negative stereotypes to problem gamblers, with the majority considering them to be impulsive, irresponsible, irrational, foolish, untrustworthy, unproductive, greedy, and anti-social. Most also considered problem gambling to be highly disruptive to the person’s life and anticipated that problem gamblers would face substantial status loss and discrimination. Thus, our current sample’s perceptions generally align with those findings and suggest that effective stigma reduction efforts, particularly those that lower negative stereotyping and prejudicial attitudes, would lower both actual and anticipated public stigma as well as self-stigma. Lowering the public stereotypes associated with problem gambling may also decrease stereotype threat, which is thought to also contribute to self-stigma (Steele, 1990).

A surprising finding in this study was that experienced stigma, measured through experienced devaluation and discrimination, was not associated with either anticipated level of public stigma or self-stigma either in the bivariate or multivariate analyses. This may be because more than half the sample reported never or rarely experiencing all types of devaluation they were asked about except for being treated as if they were dishonest. Similarly, fewer than 1 in 10 respondents reported experiencing any of the forms of discrimination surveyed, except for being denied a bank loan which was reported by about one-quarter of respondents. These low rates of experienced stigma probably reflect the tendency to keep a gambling problem well-hidden (Carroll et al., 2013; Hing et al., 2016a). Previous research has also highlighted that those with concealable stigmatized conditions are affected more by anticipated stigma than by actual experiences of being shunned, degraded or discriminated against, because they tend to hide the condition (Quinn and Chaudoir, 2009). Research with larger samples capturing more experienced stigma is needed to determine the true contribution of experienced stigma to the self-stigma of problem gambling.

Future research may also be able to overcome the limitations of the current study. These include use of a convenience rather than a representative sample of people with gambling problems, along with use of an online survey precluding those without Internet access, which may have biased results. The cross-sectional design did not allow explorations of causal relationships and alternative explanations of the results have therefore been presented above. Several measures were created for the study in the absence of existing measures and these have not been validated. However, the existing scales that were used showed good reliability.

Despite these limitations, this study has made a theoretical contribution to understanding why people with gambling problems come to hold self-stigmatizing beliefs. Our results suggest that self-stigma increases with expectations that the public applies a range of negative stereotypes to people with gambling problems, holds demeaning and discriminatory attitudes toward them, and considers them to lead highly disrupted lives. These variables have an effect both directly on self-stigma, and indirectly through heightening the anticipated level of public stigma amongst those with the condition. These findings lend weight to models that conceptualize self-stigma as an internalization of actual or anticipated public stigma (Link, 1987; Link et al., 1989; Corrigan and Watson, 2002; Watson et al., 2007; Corrigan and Rao, 2012).

## Conclusion

This study has examined predictors of anticipated public stigma and self-stigma amongst a sample of people experiencing problem gambling. It adds to the meager literature on this topic by advancing our theoretical understanding of contributors to self-stigmatizing beliefs, with perceptions of stereotyping, disruptiveness and status loss and discrimination being the most salient variables amongst those examined. It has also highlighted the importance of anticipated public stigma, that is the negative attitudes, judgments and reactions anticipated by people with gambling problems, in the formation of self-stigma – even though experiences of experienced stigma may be rare due to the concealability of problem gambling. On a practical level, the results underscore the need for stigma reduction strategies that effectively reduce demeaning stereotypes, actions and attitudes. Important avenues for research into problem gambling stigma have been identified and include longitudinal studies to explicate causal pathways, studies that examine individual characteristics that might influence vulnerability to self-stigma, comparative studies of actual and anticipated stigma, and research with larger representative samples.

## Ethics Statement

Southern Cross University Human Research Ethics Committee. Survey participants were provided with an informed consent preamble before commencing the survey and asked to tick a box to verify that they had read that information and consented to participate in the study. Because of the sensitive nature of the survey and the vulnerability of the sample (problem gamblers), an informed consent preamble warned that some questions were confronting and challenging, assured respondents of confidentiality and anonymity, and advised that respondents could withdraw their participation at any time. Each page of the survey contained contact details for Lifeline and for telephone and online gambling help services.

## Author Contributions

NH helped to conceive and design the work, organized the acquisition of data, interpreted the data, drafted the manuscript, approved the final version to be published, and agrees to be accountable for all aspects of the work. AR helped to conceive and design the work, organized the acquisition of data, analyzed the data, critically revised the manuscript for important intellectual content, approved the final version to be published, and agrees to be accountable for all aspects of the work.

## Conflict of Interest Statement

The authors’ institution received financial support for this study from the Victorian Responsible Gambling Foundation. The authors have received previous research funding from organizations that are beneficiaries of gambling, including government agencies and industry organizations and the authors declare that the research was conducted in the absence of any commercial or financial relationships that could be construed as a potential conflict of interest.
